# The role of serum procalcitonin in establishing the diagnosis and prognosis of pleural infection

**DOI:** 10.1186/s12931-017-0501-5

**Published:** 2017-02-03

**Authors:** Giles Dixon, Adriana Lama-Lopez, Oliver J. Bintcliffe, Anna J. Morley, Clare E. Hooper, Nick A. Maskell

**Affiliations:** 1 Academic Respiratory Unit, School of Clinical Sciences, University of Bristol, Bristol, BS10 5NB UK; 2 North Bristol Lung Centre, North Bristol NHS Trust, Bristol, UK; 3 Worcester Royal Hospital, Worcester, UK

**Keywords:** Sensitivity and specificity, Procalcitonin, Pleura

## Abstract

**Background:**

Bacterial pleural infection requires prompt identification to enable appropriate investigation and treatment. In contrast to commonly used biomarkers such as C-reactive protein (CRP) and white cell count (WCC), which can be raised due to non-infective inflammatory processes, procalcitonin (PCT) has been proposed as a specific biomarker of bacterial infection. The utility of PCT in this role is yet to be validated in a large prospective trial. This study aimed to identify whether serum PCT is superior to CRP and WCC in establishing the diagnosis of bacterial pleural infection.

**Methods:**

Consecutive patients presenting to a tertiary pleural service between 2008 and 2013 were recruited to a well-established pleural disease study. Consent was obtained to store pleural fluid and relevant clinical information. Serum CRP, WCC and PCT were measured. A diagnosis was agreed upon by two independent consultants after a minimum of 12 months. The study was performed and reported according to the STARD reporting guidelines.

**Results:**

80/425 patients enrolled in the trial had a unilateral pleural effusion secondary to infection. 10/80 (12.5%) patients had positive pleural fluid microbiology. Investigations for viral causes of effusion were not performed. ROC curve analysis of 425 adult patients with unilateral undiagnosed pleural effusions showed no statistically significant difference in the diagnostic utility of PCT (AUC 0.77), WCC (AUC 0.77) or CRP (AUC 0.85) for the identification of bacterial pleural infection. Serum procalcitonin >0.085 μg/l has a sensitivity, specificity, negative predictive value and positive predictive value of 0.69, 0.80, 0.46 and 0.91 respectively for the identification of pleural infection. The diagnostic utility of procalcitonin was not affected by prior antibiotic use (*p* = 0.80).

**Conclusions:**

The study presents evidence that serum procalcitonin is not superior to CRP and WCC for the diagnosis of bacterial pleural infection. The study suggests routine procalcitonin testing in all patients with unilateral pleural effusion is not beneficial however further investigation may identify specific patient subsets that may benefit.

**Trial registration:**

The trial was registered with the UK Clinical Research Network (UKCRN ID 8960). The trial was approved by the South West Regional Ethics Committee (Ethical approval number 08/H0102/11).

**Electronic supplementary material:**

The online version of this article (doi:10.1186/s12931-017-0501-5) contains supplementary material, which is available to authorized users.

## Background

Infection is a common cause of undiagnosed unilateral pleural effusion associated with high morbidity and mortality [1]. The ability to identify bacterial pleural infection in adults (hereon referred to as “pleural infection”) as the likely cause at presentation enables appropriate investigations and prompt treatment [2, 3]. In addition, this would ensure patients receive prompt inpatient treatment for pleural infection rather than undergo investigations for alternative pathologies such as suspected pleural malignancy.

Pleural infection may be indicated by symptomatology, classical radiological findings, positive blood culture, raised inflammatory markers, pleural fluid cytology or positive pleural fluid microbiology [1]. However, commonly used serum biomarkers such as C-reactive protein (CRP) and white cell count (WCC) are well known to be general markers of pro-inflammatory states. Furthermore, pleural fluid culture is negative in up to 40% of samples in patients with classical symptoms and signs [2]. Pleural fluid pH, lactate dehydrogenase (LDH) and glucose have been shown to be superior to these commonly used biomarkers in identifying pleural infection [4]. Novel biomarkers found in blood and pleural fluid such as procalcitonin (PCT), soluble triggering receptor expressed on myeloid cells (sTREM) and lipopolysaccharide-binding protein (LBP) have been studied to determine their clinical utility in pleural effusions with mixed and conflicting results [5–7].

Procalcitonin has been proposed as a specific systemic marker of bacterial infection which is not affected by viral and other non-bacterial inflammatory states [8]. Previous research has attempted to clarify the role of serum and pleural fluid PCT in identifying patients with pleural infection [5, 6]. Pleural fluid PCT has been shown to have no clinical use in pleural infection diagnosis or prognosis [6]. Lee et al. have previously presented data suggesting serum PCT may have a role in differentiating parapneumonic effusions from malignant effusions [7]. However, another small study suggested serum procalcitonin does not help differentiate benign from malignant aetiologies [9]. Whilst procalcitonin is unlikely to remove the requirement for pleural fluid sampling it is yet to be determined whether serum PCT has a role as a first-line investigation in clinical practice.

This main objective of the study was to determine whether PCT is superior to white cell count and C-reactive protein when distinguishing between pleural infection and other causes of unilateral pleural effusion in adults. The secondary objectives of the article were; 1) Does the prior use of antibiotics affect the utility of PCT in the diagnosis of unilateral pleural effusion? 2) Does a high PCT level indicate a poorer outcome in pleural infection or help predict the need for surgical intervention in patients with pleural infection? 3) Does PCT have a role in identifying patients with malignancy and a co-existing infection?

## Methods

Consecutive patients presenting to a single specialist adult tertiary pleural service with a pleural effusion were invited to consent to a prospective study between March 2008 and June 2013. All patients who consented to recruitment were included in the initial stages of the study. Radiographic (chest xray) and ultrasound appearances were used to select patients with unilateral effusions. The study received ethical approval from the South West regional ethics committee (Ethical approval number 08/H0102/11) and was registered with the UK Clinical Research Network (UKCRN ID 8960). All patients provided informed written consent to take part in the study. A full history was obtained, clinical examination was performed and blood samples were taken at the time of presentation. Clinical details were collected and blood and pleural fluid samples were stored. Further investigation was performed according to national guidelines [10]. Venous blood samples were allowed to clot at room temperature and centrifuged at 1000 g. The supernatant was then stored at −80 °C. The hospital pathology department undertook all blood tests except PCT. Baseline serum PCT was measured on stored samples by a commercially available enzyme-linked fluorescent assay as per the manufacturer’s instructions by GD, ALL, AJM and CEH (Vidas® BRAHMS PCT – BioMerieux). The assay was unable to detect PCT at levels less than 0.05 μg/l.

Antibiotic use, the need for invasive intervention, duration of hospital stay and mortality was recorded. The decision for invasive intervention including chest tube drainage was determined according to national guidelines [1]. Indications for chest tube drainage included frank pus on aspiration, positive pleural fluid microbiology and pleural pH <7.2 [1]. Patients with sepsis in association with a pleural collection despite chest tube drainage and antibiotics were referred to thoracic surgery. Thoracic surgery was carried out according to an experienced multi-disciplinary team acting on an individual patient basis. All patients had a definitive diagnosis independently verified by two consultant physicians after a minimum of 12 months of follow up. In case of disagreement a consensus was established through reappraisal of relevant investigations. Follow up at 12 months was used to allow for patients who required interval investigations to confirm the diagnosis. Patients were classified into diagnostic categories according to pre-defined criteria (see Additional file 1: Full diagnostic criteria). All data except the PCT value were available during investigation and treatment. Additionally, each consultant confirming the diagnosis had all clinical information except for the PCT value.

Statistical analysis including Receiver Operator Characteristics (ROC) analyses were performed using SPSS (IBM, Chicago, IL, USA). A *p*-value of <0.05 was considered to indicate statistical significance. The optimal cut-off values were determined for the diagnostic and prognostic ability of PCT, WCC and CRP by calculating the combined sensitivity and specificity for each value using SPSS. The highest combined sensitivity and specificity was chosen as the cut-off value. There was no hierarchy between sensitivity and specificity. The cut-off value of <0.05 μg/l was also analysed when PCT was used to indicate infection. Independent samples *T*-test was used to compare PCT, WCC and CRP in certain patient subsets.

The study used the STARD reporting guidelines (see Additional file 2: STARD reporting guidelines) [11].

## Results

### Study population demographics

**Fig. 1:**
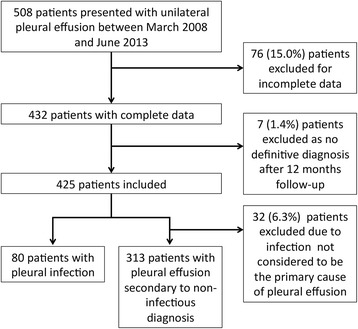
Flow chart showing the selection of patients for inclusion in the study

Five hundred and eight patients with a unilateral pleural effusion were recruited. 432 patients had a complete data set available for analysis. Full results were unavailable for 76 (15.0%) patients due to inadequate sample storage. The diagnoses of these patients can be found in Table 1. A definitive diagnosis could not be established for 7 patients (1.4%). Therefore 425 (83.7%) patients were analysed in this study (Fig. 1). The median age and interquartile range, sex ratio, patient setting and diagnosis for all 508 patients can be found in Table 1. The 12-month diagnoses of the 425 patients analysed in the study along with the median PCT, WCC and CRP with interquartile range can be found in Table 2.

**Table 1:** Patient characteristics for 508 patients recruited to study between March 2008 and June 2013

		Patients included in analysis (*n* = 425)	Patients excluded from analysis for incomplete data (*n* = 76)	Patients excluded from analysis for no definitive diagnosis (*n* = 7)
Male:female		283:142 (67:33%)	51:25 (67:33%)	7:0 (100:0%)
Median age (IQR)		72 (63–80)	75 (69–78)	81 (55.5–88.5)
Inpatient:outpatient		179:246 (42:58%)	36:39 (1 data not available) (47:51%)	5:2 (71:29%)
Diagnosis	Metastatic malignancy	169 (39.8%)	32 (42.1%)	–
	Pleural infection	80 (18.8%)	11 (14.5%)	–
	Malignant mesothelioma	70 (16.5%)	4 (5.3%)	–
	Congestive cardiac failure	41 (9.6%)	7 (9.2%)	–
	BAPE	17 (4.0%)	6 (7.9%)	–
	Inflammatory pleuritis	13 (3.1%)	7 (9.2%)	–
	Pleural TB	10 (2.4%)	0	–
	Other	25 (5.9%)	9 (11.8%)	–

Diagnoses classed as “Other” included; renal failure, hepatic hydrothorax, pulmonary embolism, iatrogenic, trauma, drug-induced, connective tissue disease, post coronary artery bypass graft, pancreatitis and Meigs syndrome

*Abbreviations*: *BAPE* benign asbestos related pleural effusion, *IQR* interquartile range, *TB* tuberculosis

**Table 2:** Twelve-month diagnosis of 425 patients presenting with unilateral pleural effusion between 2008 and 2013 and the median and interquartile range of serum procalcitonin, white cell count and C-reactive protein

Diagnosis	*n*	%	Procalcitonin (μg/l)	White cell count (×10^9^/L)	C-reactive protein (mg/l)
Metastatic malignancy	169	39.8%	<0.05 (0–0.12)	8.9 (7.4–11.0)	34.5 (12–90.75)
Pleural infection	80	18.8%	0.2 (0.1–0.7)	12.5 (9.2–16.3)	135.3 (71.6–239.6)
Malignant mesothelioma	70	16.5%	<0.05 (<0.05- < 0.05)	8.7 (7.1–9.7)	22.2 (9.5–70.3)
Congestive cardiac failure	41	9.6%	<0.05 (<0.05–0.1)	7.4 (6.2–9.2)	9.0 (5.1–22.6)
BAPE	17	4.0%	<0.05 (<0.05–0.1)	8.5 (7.5–9.4)	27.0 (15.4–53.0)
Inflammatory pleuritis	13	3.1%	<0.05 (<0.05–0.1)	7.1 (6.3–7.9)	10.0 (5.0–48.0)
Pleural TB	10	2.4%	0.1 (<0.05–0.2)	7.1 (6.9–7.5)	76.5 (36.9–111.7)
Renal failure	6	1.4%	0.1 (0.1–0.2)	6.8 (5.8–10.3)	25.0 (20.8–37.3)
Hepatic hydrothorax	4	0.9%	<0.05 (<0.05–0.1)	5.0 (4.2–7.5)	2.0 (2.0–4.7)
Pulmonary embolism	3	0.7%	<0.05 (n/a)	5.8 (n/a)	14.0 (n/a)
Iatrogenic/Trauma	3	0.7%	<0.05 (n/a)	10.4 (n/a)	32 (n/a)
Connective tissue disease	3	0.7%	<0.05 (n/a)	9.0 (n/a)	29.0 (n/a)
Post-CABG	3	0.7%	<0.05 (n/a)	7.6 (n/a)	3.0 (n/a)
Pancreatitis	2	0.5%	<0.05 (n/a)	4.0 (n/a)	19.0 (n/a)
Drug-induced	1	0.2%	<0.05 (n/a)	8.6 (n/a)	13 (n/a)

*Abbreviations*: *BAPE* benign asbestos related pleural effusion, *CABG* coronary artery bypass graft, *IQR* interquartile range, *TB* tuberculosis

Eighty patients had a 12-month diagnosis of effusion secondary to pleural infection.

### Does serum procalcitonin provide greater diagnostic utility over white cell count and C-reactive protein in the diagnosis of pleural infection?

The main objective of the study was to assess whether PCT had a greater diagnostic utility over WCC and CRP for the identification of pleural infection. Pleural infection was diagnosed in 80/425 patients. 40 patients were diagnosed following a computerised tomography (CT) scan in keeping with infection with radiological resolution following treatment with antibiotics, 23 patients had a pleural fluid pH < 7.2 and clinical presentation suggestive of sepsis, 10 patients with pleural infection had positive pleural fluid microbiology, 6 patients had frank pus on pleural aspiration and 1 patient was diagnosed on histology.

**Fig. 2:**
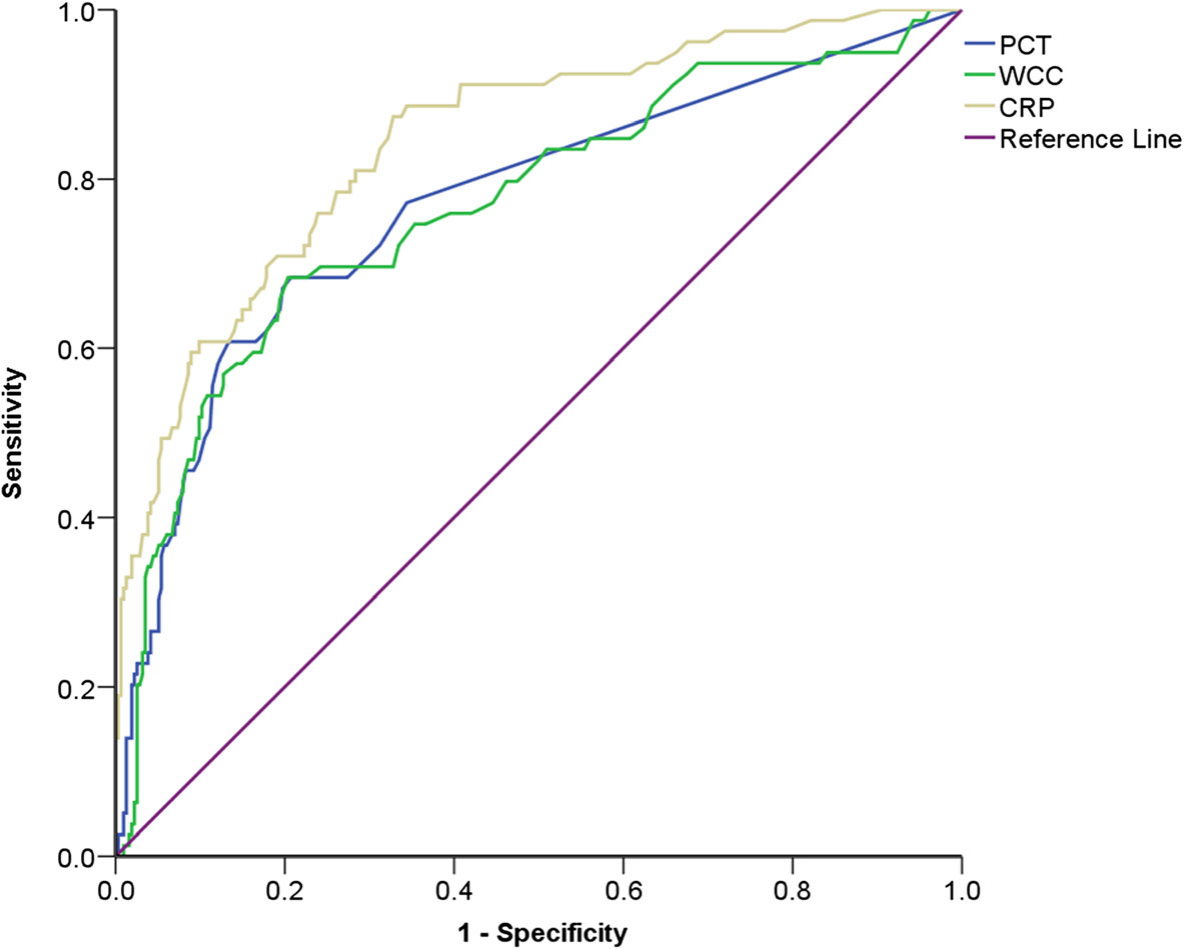
ROC curve to show the ability of serum procalcitonin, white cell count and C-reactive protein to distinguish infective vs non-infective causes of unilateral pleural effusion

32/345 patients without a primary diagnosis of pleural infection had an infection which was not considered to be the primary cause of unilateral pleural effusion. Of these 22 patients had pneumonia (contralateral or not temporally related to the effusion), 6 patients had a urinary tract infection, 1 patient had ophthalmic shingles, 1 patient had overwhelming sepsis of no clear source, 1 patient had a septic joint and 1 patient had a sub-phrenic abscess. Those patients with a co-existing infection were excluded from analysis. ROC curve analysis showed PCT, WCC and CRP had an area under the curve (AUC) of 0.77 (95% CI = 0.71–0.84), 0.77 (95% CI = 0.71–0.83) and 0.85 (0.80–0.90) respectively for identifying patients with pleural infection (Fig. 2). Procalcitonin did not exhibit diagnostic characteristics superior to either WCC or CRP (Table 3).

**Table 3:** The sensitivity, specificity, positive predictive value and negative predictive value of PCT, WCC and CRP in the diagnosis of pleural infection

Serum biomarker			Sensitivity	Specificity	Positive predictive value	Negative predictive value	Positive likelihood ratio	Negative likelihood ratio
PCT	AUC calculated optimal cut-off value	≥0.085 μg/l	0.69	0.80	0.46	0.91	3.36	0.39
	Manufacturers recommended cut-off value	≥0.05 μg/l	0.78	0.66	0.37	0.92	2.27	0.34
WCC	AUC calculated optimal cut-off value	≥10.35x10^9^/L	0.69	0.80	0.46	0.91	3.36	0.39
CRP	AUC calculated optimal cut-off value	≥46.5 mg/l	0.88	0.67	0.41	0.95	2.69	0.19

*Abbreviations*: *AUC* area under the curve, *CRP* C reactive protein, *PCT* procalcitonin, *WCC* white cell count

24/425 patients had a low CRP (<46.5 mg/l) and a low WCC (<10.35 × 10^9^/L) and a high PCT ≥0.085 μg/l. Two/twenty-four of these patients had a diagnosis of pleural infection. The causes of effusion in the remaining patients were metastatic malignancy (6), congestive cardiac failure (6), renal failure (3), inflammatory pleuritis (2), BAPE (1), hepatic hydrothorax (1), malignant mesothelioma (1) iatrogenic (1) and pleural tuberculosis (1). 1 patient had a concurrent urinary tract infection.

### Does the use of antibiotics prior to presentation significantly affect the diagnostic utility of procalcitonin, white cell count and C-reactive protein?

**Fig. 3:**
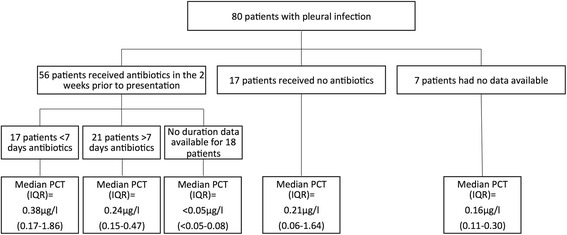
The effect of prior use of antibiotics on the diagnostic utility of procalcitonin in 80 patients presenting to the pleural service with pleural infection

Eighty patients had a 12-month diagnosis of pleural infection and of these 56 patients had received antibiotics prior to presentation to the pleural service, 17 patients had no prior antibiotics and data was unavailable in 7 cases. The duration of antibiotic use is outlined in Fig. 3.

There was no statistically significant difference between PCT (*p* = 0.80), WCC (*p* = 0.16) and CRP (*p* = 0.56) in the patient group who received antibiotics and those who did not (Additional file 3: Additional dataset 1). Furthermore there was no significant statistical difference between those who received antibiotics for ≥7 days and those who did not receive antibiotics (*p* = 0.48).

### Does procalcitonin provide relevant prognostic information in pleural infection?

**Fig. 4:**
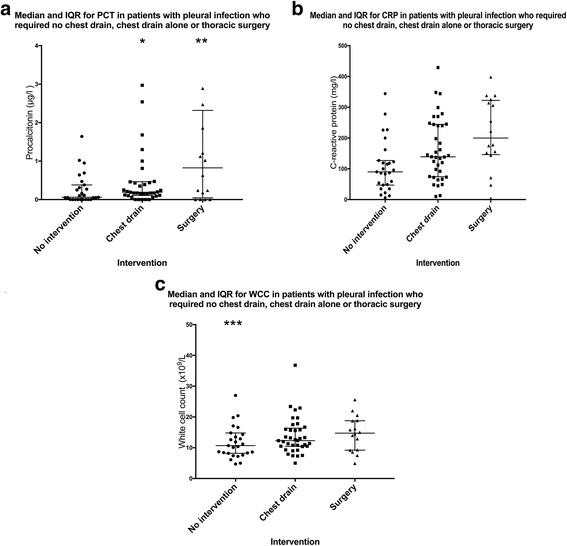
Dot plots showing median and IQR of PCT, WCC and CRP in patients who received no intervention, chest drain insertion or thoracic surgery for pleural infection. Outlier values; * 7.5, 14.68, ** 7.55, 45.49, *** 62.0. *CRP = C-reactive protein, IQR = Interquartile range, PCT = Procalcitonin, WCC = White cell count*

Patient outcomes and interventions were recorded for 80 cases of pleural infection. 27 patients required no chest drain, 37 patients required a chest drain alone and 16 patients required thoracic surgery. The median and interquartile range of PCT, CRP and WCC for patients who required no chest drain, chest drain alone or thoracic surgery can be found in Fig. 4 as a dot plot.

ROC curve analysis showed PCT, WCC and CRP had an AUC (95% CI) of 0.65 (0.48–0.83), 0.60 (0.44–0.76) and 0.69 (0.54–0.85) respectively for the ability to predict the need for surgical intervention vs chest drain or no intervention (Additional file 4: ROC curve analysis of PCT, WCC and CRP for the ability to predict the need for surgical intervention vs chest drain or no intervention in patients with pleural infection). PCT did not show greater prognostic ability than CRP and WCC for the identification of patient who required thoracic surgery (See Additional file 3: Additional dataset 2).

Procalcitonin >1.01 μg/l showed a sensitivity, specificity, positive predictive value and negative predictive value of 0.50, 0.88, 0.50 and 0.88 respectively for the identification of those patients who would require surgical intervention.

12/80 patients died within 1 year of presentation with pleural infection. 3/12 patients had a known malignancy not thought to be the primary cause of their unilateral pleural effusion and 4 patients had significant co-morbidities including atrial fibrillation, ischaemic heart disease and heart failure. Procalcitonin (*p* = 0.92), WCC (*p* = 0.50) and CRP (*p* = 0.17) were not significantly higher in the group of patients who died within 1 year of presentation.

### Is procalcitonin superior to white cell count and C-reactive protein biomarkers in identifying patients with malignancy and a co-existing pleural infection?

Two-hundred and thirty nine patients with a 12-month diagnosis of pleural malignancy were analysed (malignant mesothelioma or metastatic malignancy) to assess whether PCT could accurately predict the presence of co-existing infection. 23/239 patients presented with a concurrent bacterial infection; 18 with pneumonia, 3 with a UTI, 1 with sepsis and 1 with ophthalmic shingles with bacterial infection. ROC curve analysis for the ability of PCT, WCC and CRP to predict the presence of concurrent bacterial infection showed an AUC (95% CI) of 0.68 (0.56–0.80), 0.68 (0.55–0.80) and 0.64 (0.50–0.77) respectively (Additional file 5: ROC curve analysis of PCT, WCC and CRP for the ability to predict presence of co-existing bacterial infection in patients presenting with unilateral pleural effusion secondary to malignancy). There was no statistically significant difference between PCT for those patients with or without a co-existing bacterial infection (Table 4). WCC and CRP were significantly increased in patients with a co-existing infection. PCT showed no significant greater diagnostic utility over WCC or CRP for ability to distinguish between those patients with pleural malignancy and co-existing infection and those without co-existing infection (See Additional file 3: Additional dataset 3).

**Table 4:** Median and IQR of PCT, WCC and CRP for patients with pleural malignancy with and without a co-existing bacterial infection

	Co-existing infection	No co-existing infection	*p*-value
*N*	23	216	–
PCT (Median (IQR)) μg/l	0.11 (<0.05–0.15)	<0.05 (<0.05–0.08)	0.75
WCC (Median (IQR)) ×10^9^/L	10.0 (8.5–20.3)	8.7 (7.2–10.2)	0.004
CRP (Median (IQR)) mg/l	88.3 (11.4–135.8)	29.0 (11.0–81.5)	0.0003

## Discussion

This trial is the largest prospective analysis of serum PCT measurement in patients presenting with unilateral pleural effusions. We have demonstrated that PCT when measured in all patients presenting with unilateral pleural effusion to a tertiary pleural service offers no greater diagnostic utility than WCC or CRP. Furthermore we have found that PCT has no significant prognostic value in pleural infection in terms of predicting 1 year mortality or need for thoracic surgical intervention. CRP and WCC were significantly higher in patients with pleural malignancy who had a co-existing bacterial infection.

Procalcitonin is a peptide precursor for calcitonin which is released by the C-cells of the thyroid gland. Under normal conditions levels of circulating procalcitonin are negligible. Procalcitonin has been studied in a number of scenarios where it has been presented as a test for distinguishing between bacterial infection and other inflammatory insults [8]. Procalcitonin can distinguish between bacterial and aseptic meningitis, identify infectious vs non-infectious exacerbations of chronic obstructive pulmonary disease (COPD) and help predict aetiology and outcome in community acquired pneumonia [9, 12–14]. Procalcitonin has been studied in pleural disease both in the pleural fluid and serum however this is the first study to attempt to characterise the role of serum PCT as an immediate “bed-side” test in all patients presenting with an undiagnosed unilateral effusion.

Signs of pleural infection and pleural inflammation can often be similar and can pose diagnostic difficulties. Commonly used biomarkers such as CRP and WCC are markers of inflammation and can be raised indiscriminately in pro-inflammatory states such as malignancy and infection [10]. Previously pleural fluid PCT has been shown to be inferior to a range of other markers in distinguishing between infected and non-infected effusions as well as being inferior to serum PCT [6, 7, 15, 16]. Serum PCT studies have suggested that higher values could be a useful indicator of pleural infection, however only specifically selected small numbers of patients (<100) have been studied [15]. McCann et al. addressed the issue of inflammation vs infection by comparing PCT and CRP levels before and after a sterile inflammatory insult (talc pleurodesis). The study found that whilst CRP rose significantly (360%) the rise in PCT was less marked (21%); however, this is just one cause of non-infective inflammation and it is yet to be determined whether malignant processes have the same effect on PCT.

We analysed serum PCT, WCC and CRP in consecutive patients presenting to our tertiary referral centre prospectively recruited to a pleural study. Procalcitonin has previously been presented as a marker of infection which can be used to guide the duration of antibiotic therapy [17, 18]. Therefore it was predicted that antibiotic therapy may reduce the ability of PCT to identify infection. In our cohort, 68% of patients (56/80) with pleural infection had received antibiotics in the 2 weeks preceding presentation. We have shown that the use of antibiotics did not significantly affect the diagnostic utility of procalcitonin. It could be suggested that a longer duration of antibiotics (≥7 days) prior to investigation could theoretically hinder the diagnostic ability of procalcitonin. However, we have shown no statistically significant difference in procalcitonin between those patients who received ≥7 days of antibiotics and those who received none (*p* = 0.48).

The diagnostic ability of PCT in the presence of renal failure has not been conclusively verified. Previous studies have suggested that renal function may interfere with PCT values [19, 20]. We found patients with unilateral pleural effusions secondary to renal failure with no evidence of infection had raised levels of PCT (Table 2). However, only 6 patients were diagnosed with a unilateral pleural effusion secondary to renal failure. Further studies are required to assess the relationship between renal failure and PCT in patients with unilateral pleural effusions.

Predicting outcome in pleural infection is difficult, it has previously been shown there are no reliable clinical, pleural fluid or radiological characteristics that will predict failure of medical therapy upon admission [21]. One of the key diagnostic questions is how to identify those patients who require invasive intervention such as pleural drainage or thoracic surgery. Persistently high inflammatory markers such as CRP and WCC have been shown to be associated with poorer outcomes and the need to perform chest tube drainage [22]. Around 30% of patients can be expected to need thoracic surgery or die as a result of pleural infection [23]. Currently, there is no defined criteria for surgical intervention in pleural infection and the decision to operate remains subjective [1]. Our study has shown no statistically significant difference in PCT, WCC and CRP between patients who required no intervention, chest tube drainage or thoracic surgery. This is in line with previously reported conclusion using the Multicentre Intra-pleural Sepsis Trial (MIST) data [5]. Fig. 4 does show a strong trend between PCT and degree of intervention required and increased sample size may allow sufficient power to identify a statistically significant difference.

Unilateral pleural effusions can have dual pathology with a number of processes contributing to the accumulation of fluid. Pleural malignancy may be complicated by either intra- or extra-thoracic infection. Identifying those patients who may require antibiotics despite a primary malignant process could improve short-term outcomes. Approximately 10% (23/239) of patients with a diagnosis of malignant unilateral pleural effusion also had a co-existing infection at presentation. Our data shows PCT has no significant greater diagnostic utility over WCC or CRP in identifying patients who have pleural infection alongside a malignant process. Furthermore CRP and WCC were found to be significantly higher in patients with a co-existing bacterial infection (*p* = 0.004 and *p* = 0.0003 respectively).

### Limitations

There are limitations to the current study. The study initially investigated the role of PCT in all patients presenting to a pleural service and then focussed on specific subsets of patients. Whilst the current study failed to identify a specific subset where PCT out-performed WCC and CRP there may be subsets not yet analysed who may benefit from serum PCT analysis. Further studies to specifically select patients at presentation who may benefit from PCT measurement could identify a diagnostic role for serum PCT.

The analysis was intended to look at the diagnostic capabilities in all patients attending the pleural service however 76/508 patients were excluded because of insufficient storage of sample fluid. There were 7 patients excluded from the trial due to no definitive diagnosis. Further investigation of these patients was not carried out due to clinical resolution of symptoms. The number of patients not consenting to trial inclusion was not recorded. The gold standard for the diagnosis of pleural infection is positive pleural fluid microbiology. In our study 10/80 (12.5%) patients had positive pleural fluid microbiology, this is lower than reported culture positive rates and may be because of the high numbers of patients who had received antibiotics prior to their presentation to our tertiary centre [23, 24]. Therefore in order to diagnose infection we relied upon other classical markers such as low pleural fluid pH. Further study of patients with culture-positive pleural infection is required to validate the results.

Pleural effusion may also be caused by viral infection [25]. Procalcitonin is a poor diagnostic marker of viral infection and therefore may partly explain the current findings.

The study was conducted at a single centre, and the results may not be representative of patient subgroups in other healthcare settings, particularly with respect to decision-making around invasive procedures and surgery specifically, the thresholds for which may vary internationally.

In our study PCT was only measured at one time point (at initial presentation). Previous studies in non-pleural disease have used procalcitonin as a biomarker to decide when to stop antibiotic therapy [17, 18]. The role of PCT in monitoring response to treatment was not assessed by the current study.

The measurement of serum PCT was performed on frozen stored samples of serum. We were unable to guarantee the samples had not been altered through the storage process. The study design meant that the investigators and clinicians were not blinded to CRP or WCC during treatment and diagnosis and these values may have influenced decisions on management and specifically thoracic surgery. Furthermore, the CRP and WCC may have influenced the definitive consultant diagnosis thus overestimating the diagnostic accuracy of CRP and WCC. The PCT value, however, was not available during investigation, treatment or diagnosis.

Procalcitonin has previously been used as a marker of systemic infection [8]. The intention of the study was to assess the diagnostic utility of PCT in pleural infection rather than its use as a marker of systemic infection. It is possible that PCT failed to outperform traditional biomarkers because compartmentalised pleural infection has a reduced effect on serum PCT.

To prevent analysis of patients with raised PCT due to non-pleural infection we excluded patients with infections that were not the primary cause of the unilateral pleural effusion from our analysis. However in practice the location of infection may not always be apparent at presentation and as such further targeted investigations are always required.

Our study attempted to analyse the effect of the prior use of antibiotics on the diagnostic capabilities of PCT. The study was limited as just 17 patients received no antibiotics prior to presentation. It is likely that PCT responds to antibiotics in 24–48 h and therefore further analysis should be carried out on patients who had received <48 h of antibiotics. The current dataset would have to be extended to confirm the relationship between antibiotic use and serum PCT.

## Conclusions

In summary this large study provides evidence that there is no greater diagnostic utility in the routine use of serum PCT in adult patients presenting with unilateral pleural effusions. No additional diagnostic utility was found when using procalcitonin over CRP and WCC in identifying bacterial pleural infection. The current study also suggests PCT also offers no significant prognostic information in pleural infection with respect to predicting the need for surgery or mortality. This study should discourage the routine use of serum PCT all patents presenting with unilateral pleural effusion. However further studies may identify specific subsets of patients who may benefit from PCT measurement.

## Additional files

Additional file 1: Full diagnostic criteria. (DOCX 13 kb)
Additional file 2: STARD reporting guidelines. (DOC 48 kb)
Additional file 3: Additional dataset 1–3. (DOCX 63 kb)
Additional file 4: ROC curve analysis of PCT, WCC and CRP for the ability to predict the need for surgical intervention vs chest drain or no intervention in patients with pleural infection. (TIFF 226 kb)
Additional file 5: ROC curve analysis of PCT, WCC and CRP for the ability to predict presence of co-existing bacterial infection in patients presenting with unilateral pleural effusion secondary to malignancy. (TIF 380 kb)

## Abbreviations

AUC: Area under curve
BAPE: Benign asbestos related pleural effusion
CABG: Coronary artery bypass graft
CI: Confidence interval
COPD: Chronic obstructive pulmonary disease
CRP: C-reactive protein
CT: Computerised tomography
IQR: Interquartile range
LBP: Lipopolysaccharide-binding protein
LDH: Lactate dehydrogenase
MIST: Multicentre intra-pleural sepsis trial
PCT: Procalcitonin
ROC: Receiver operator characteristics
STREM: Soluble triggering receptor expressed on myeloid cells
TB: Tuberculosis
WCC: White cell count
