# Supplementary material for: The role of serum procalcitonin in establishing the diagnosis and prognosis of pleural infection
Source: Respir Res. 2017 Feb 3;18:30. doi: 10.1186/s12931-017-0501-5 (PMC5291982; doi:10.1186/s12931-017-0501-5)
Supplement: Additional file 1: — Full diagnostic criteria. (DOCX 13 kb) [file 12931_2017_501_MOESM1_ESM.docx]

**Supporting information 1**

**Diagnostic criteria for identifying the cause of pleural effusion**

**Metastatic malignancy**

Malignant pleural fluid cytology *or* biopsy *or* histologically confirmed extra-thoracic/pulmonary malignancy with radiographic evidence of metastasis to ipsilateral pleura on CT *or* radiological changes which have progressed in keeping with malignancy on interval CT scan in the correct clinical context *or* autopsy confirming malignancy

**Malignant mesothelioma**

Diagnostic pleural biopsy or cytology with CT appearances in keeping with primary pleural malignancy

**Pleural infection**

**Complicated Parapneumonic Effusion**

Clinical presentation suggestive of sepsis *and* pleural fluid pH ≤7.2 *or* pleural fluid loculation on ultrasound *and* follow up for at least 6months inconsistent with pleural malignancy *or* pleural fluid gram stain *or* culture positive *or* frank pus *or* pleural infection confirmed by pleural biopsy histology *and*/*or* microbiological culture *or* CT scan consistent with pleural infection with radiological resolution following treatment with antibiotics.

**Simple Parapneumonic effusion**

Clinical presentation suggestive of sepsis with appropriate chest radiology *and* pleural fluid which is gram stain *and* culture negative with a pH >7.2 *and* an absence of loculation on thoracic ultrasound *and* resolution of effusion on CXR after antibiotics *or* clinical progression to pleural infection (see above)

**Congestive Cardiac Failure**

History *and* examination features of CCF *or* evidence of at least moderate left ventricular systolic *or* diastolic failure *or* severe valvular disease on echo *or* improvement of effusion *and* symptoms with diuretic therapy

**Inflammatory Pleuritis**

Demonstration of non-specific inflammatory pleuritis on pleural biopsy *and* follow-up for 12 months without progression that would suggest a malignant cause.

**Benign Asbestos Related Pleural Effusion**

History of asbestos exposure *or* evidence of pleural plaques on CT *and* stable *or* improving CT appearances with follow-up for at least 12 months. (The development of enfolded lung is allowed) *or* negative thoracoscopy (benign pleural biopsy)

**Renal failure**

Biochemical confirmation of renal failure in the absence of clinical, radiological *or* pleural fluid analysis suspicious of an alternative cause.

**Pleural Tuberculosis**

Culture *or* AAFB positive sputum, pleural fluid *or* pleural tissue *and* resolution of pleural effusion with anti TB therapy at 6 month follow-up.

**Undiagnosed**

Exhaustive investigations including 12 months follow-up with interval CT scans has not demonstrated a diagnosis *or* patient unfit for further investigation *and* follow up *or* patient died without definitive diagnosis *and* no post mortem examination conducted

**Hepatic hydrothorax**

Known history *or* clinical presentation consistent with liver disease *and* recurrent transudative pleural effusion *and* negative cytology

**Pulmonary embolism**

Evidence of PE on CT pulmonary arteriography and no alternative explanation for pleural effusion on cross sectional imaging *or* pleural fluid analysis.

**Connective tissue disease (including Rheumatoid Arthritis)**

Systemic features *or* known diagnosis of connective tissue disease *and* chest radiology (including CT imaging) showing benign features (e.g. doesn’t meet any of Leung’s criteria) with at least 6 months follow-up *and* /*or* pleural biopsy negative for malignancy.

**Coronary Artery Bypass Graft (CABG)**

CABG in 3 months prior to development of pleural effusion in the absence of an alternative cause

**Drug**

Patient received a drug that is recognised to cause pleural effusions with complete resolution upon withdrawal of the drug *and* no clear alternative diagnosis

**Iatrogenic/Trauma**

Iatrogenic/traumatic event that lead to a pleural effusion *and* no clear alternative diagnosis

**Pancreatitis**

Raised amylase *or* clinical signs and symptoms in keeping with pancreatitis *and* no clear alternative diagnosis
