# Supplementary material for: The role of serum procalcitonin in establishing the diagnosis and prognosis of pleural infection
Source: Respir Res. 2017 Feb 3;18:30. doi: 10.1186/s12931-017-0501-5 (PMC5291982; doi:10.1186/s12931-017-0501-5)
Supplement: Additional file 3: — Additional dataset 1–3. (DOCX 63 kb) [file 12931_2017_501_MOESM3_ESM.docx]

**S3. Supporting information 3. Additional datasets**

**Supporting information 3. Additional dataset 1.** Median and IQR for PCT, WCC and CRP of patients with pleural infection who had and had not received antibiotics prior to presentation

|  | Antibiotics | No antibiotics | No information |
| --- | --- | --- | --- |
| N | 56 | 17 | 7 |
| PCT (Median (IQR)) µg/l | 0.19(<0.05-0.47) | 0.21 (0.06-1.64) | 0.16 (0.11-0.30) |
| WCC (Median (IQR)) x10^9^/L | 12.0 (9.2-15.7) | 12.3 (8.6-18.8) | 15.0 (13.8-18.8) |
| CRP (Median (IQR)) mg/l | 130.5 (72.5-223.1) | 138.0 (83.0-240.0) | 199.9 (20.6-333.8) |

*CRP = C reactive protein, IQR = interquartile range, PCT = procalcitonin, WCC = white cell count.*

**Supporting information 3. Additional dataset 2.** The sensitivity, specificity, positive predictive value and negative predictive value of PCT, WCC and CRP for the ability to distinguish between those patients with pleural infection who required thoracic surgery and those required chest drain alone or no intervention

| Serum biomarker | Optimal cut-off value | Sensitivity | Specificity | Positive predictive value | Negative predictive value | Positive likelihood ratio | Negative likelihood ratio |
| --- | --- | --- | --- | --- | --- | --- | --- |
| PCT | ≥1.01µg/l | 0.50 | 0.88 | 0.50 | 0.88 | 4.00 | 0.57 |
| WCC | ≥13.75x10^9^/L | 0.63 | 0.67 | 0.32 | 0.88 | 1.90 | 0.56 |
| CRP | ≥143.5mg/l | 0.81 | 0.64 | 0.36 | 0.93 | 2.26 | 0.29 |

*CRP = C reactive protein, PCT = procalcitonin, WCC = white cell count.*

**Supporting information 3. Additional dataset 3.** The sensitivity, specificity, positive predictive value and negative predictive value of PCT, WCC and CRP for the ability to distinguish between those patients with pleural malignancy and co-existing infection and those without co-existing infection

| Serum biomarker |  |  | Sensitivity | Specificity | Positive predictive value | Negative predictive value | Positive likelihood ratio | Negative likelihood ratio |
| --- | --- | --- | --- | --- | --- | --- | --- | --- |
| PCT | AUC calculated optimal cut-off value | ≥0.065µg/l | 0.65 | 0.73 | 0.20 | 0.95 | 2.39 | 0.48 |
|  | Manufacturers recommended cut-off value | ≥0.05µg/l | 0.65 | 0.67 | 0.17 | 0.95 | 1.98 | 0.52 |
| WCC | AUC calculated optimal cut-off value | ≥15.35x10^9^/L | 0.39 | 0.95 | 0.47 | 0.94 | 8.45 | 0.64 |
| CRP | AUC calculated optimal cut-off value | ≥115mg/l | 0.43 | 0.87 | 0.26 | 0.94 | 3.24 | 0.65 |

*AUC = Area under the curve, CRP = C reactive protein, PCT = procalcitonin, WCC = white cell count.*
