# Supplementary figures and images for: The role of serum procalcitonin in establishing the diagnosis and prognosis of pleural infection
Source: Respir Res. 2017 Feb 3;18:30. doi: 10.1186/s12931-017-0501-5 (PMC5291982; doi:10.1186/s12931-017-0501-5)

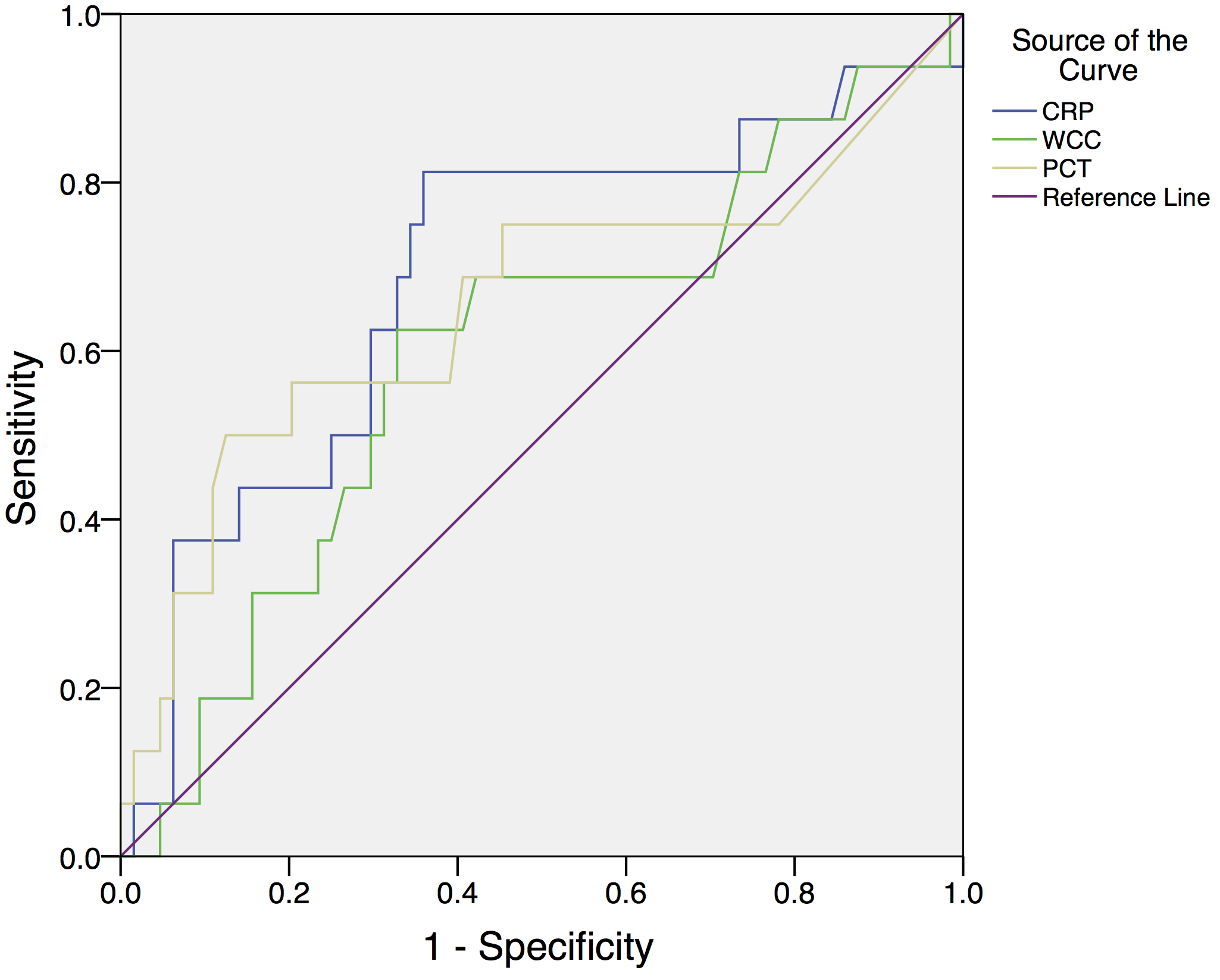

Supplement: Additional file 4: — ROC curve analysis of PCT, WCC and CRP for the ability to predict the need for surgical intervention vs chest drain or no intervention in patients with pleural infection. (TIFF 226 kb) [file 12931_2017_501_MOESM4_ESM.tiff]

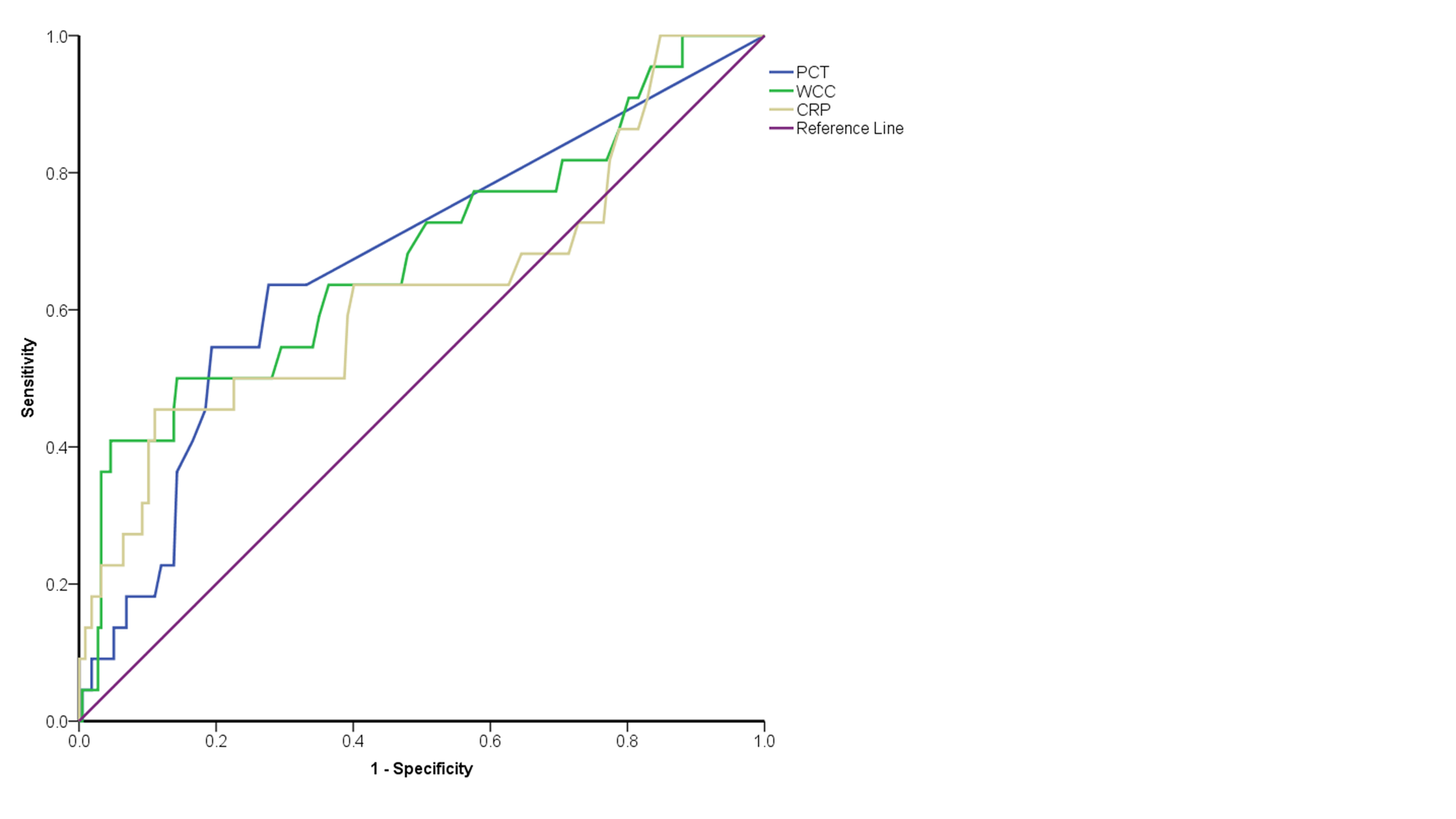

Supplement: Additional file 5: — ROC curve analysis of PCT, WCC and CRP for the ability to predict presence of co-existing bacterial infection in patients presenting with unilateral pleural effusion secondary to malignancy. (TIF 380 kb) [file 12931_2017_501_MOESM5_ESM.tif]
